# Machine learning patterns for neuroimaging-genetic studies in the cloud

**DOI:** 10.3389/fninf.2014.00031

**Published:** 2014-04-08

**Authors:** Benoit Da Mota, Radu Tudoran, Alexandru Costan, Gaël Varoquaux, Goetz Brasche, Patricia Conrod, Herve Lemaitre, Tomas Paus, Marcella Rietschel, Vincent Frouin, Jean-Baptiste Poline, Gabriel Antoniu, Bertrand Thirion, IMAGEN Consortium

**Affiliations:** ^1^Parietal Team, INRIA Saclay, Île-de-FranceSaclay, France; ^2^CEA, DSV, I^2^BM, NeurospinGif-sur-Yvette, France; ^3^KerData Team, INRIA Rennes - Bretagne AtlantiqueRennes, France; ^4^Microsoft, Advance Technology Lab EuropeMunich, Germany; ^5^Institute of Psychiatry, King's College LondonLondon, UK; ^6^Department of Psychiatry, Universite de Montreal, CHU Ste Justine HospitalMontreal, QC, Canada; ^7^Institut National de la Santé et de la Recherche Médicale, INSERM CEA Unit 1000 “Imaging & Psychiatry,” University Paris Sud, Orsay, and AP-HP Department of Adolescent Psychopathology and Medicine, Maison de Solenn, University Paris DescartesParis, France; ^8^Rotman Research Institute, University of TorontoToronto, ON, Canada; ^9^School of Psychology, University of NottinghamNottingham, UK; ^10^Montreal Neurological Institute, McGill UniversityMontréal, QC, Canada; ^11^Central Institute of Mental HealthMannheim, Germany; ^12^Medical Faculty Mannheim, University of HeidelbergHeidelberg, Germany; ^13^Henry H. Wheeler Jr. Brain Imaging Center, University of California at BerkeleyBerkeley, CA, USA; ^14^http://www.imagen-europe.com/

**Keywords:** machine learning, neuroimaging-genetic, cloud computing, fMRI, heritability

## Abstract

Brain imaging is a natural intermediate phenotype to understand the link between genetic information and behavior or brain pathologies risk factors. Massive efforts have been made in the last few years to acquire high-dimensional neuroimaging and genetic data on large cohorts of subjects. The statistical analysis of such data is carried out with increasingly sophisticated techniques and represents a great computational challenge. Fortunately, increasing computational power in distributed architectures can be harnessed, if new neuroinformatics infrastructures are designed and training to use these new tools is provided. Combining a MapReduce framework (TomusBLOB) with machine learning algorithms (Scikit-learn library), we design a scalable analysis tool that can deal with non-parametric statistics on high-dimensional data. End-users describe the statistical procedure to perform and can then test the model on their own computers before running the very same code in the cloud at a larger scale. We illustrate the potential of our approach on real data with an experiment showing how the functional signal in subcortical brain regions can be significantly fit with genome-wide genotypes. This experiment demonstrates the scalability and the reliability of our framework in the cloud with a 2 weeks deployment on hundreds of virtual machines.

## 1. Introduction

Using genetics information in conjunction with brain imaging data is expected to significantly improve our understanding of both normal and pathological variability of brain organization. It should lead to the development of biomarkers and in the future personalized medicine. Among other important steps, this endeavor requires the development of adapted statistical methods to detect significant associations between the highly heterogeneous variables provided by genotyping and brain imaging, and the development of software components with which large-scale computation can be done.

In current settings, neuroimaging-genetic datasets consist of a set of (1) genotyping measurements at given genetic loci, such as Single Nucleotide Polymorphisms (SNPs) that represent a large amount of the genetic between-subject variability, and (2) quantitative measurements at given locations (voxels) in three-dimensional images, that represent e.g., either the amount of functional activation in response to a certain task or an anatomical feature, such as the density of gray matter in the corresponding brain region. These two sets of features are expected to reflect differences in brain organization that are related to genetic differences across individuals.

Most of the research efforts so far have been focused on designing association models, while the computational procedures used to run these models on actual architectures have not been considered carefully. Voxel intensity and cluster size methods have been used for genome-wide association studies (GWAS) (Stein et al., 2010), but the multiple comparisons problem most often does not permit to find significant results, despite efforts to estimate the effective number of tests (Gao et al., 2010) or by paying the cost of a permutation test (Da Mota et al., 2012). Working at the genes level instead of SNPs (Hibar et al., 2011; Ge et al., 2012) is a promising approach, especially if we are looking at monogenic (or few causal genes) diseases.

For polygenic diseases, gains in sensitivity might be provided by multivariate models in which the joint variability of several genetic variables is considered simultaneously. Such models are thought to be more powerful (Meinshausen and Bühlmann, 2010; Vounou et al., 2010; Bunea et al., 2011; Kohannim et al., 2011; Floch et al., 2012), because they can express more complex relationships than simple pairwise association models. The cost of unitary fit is high due to high-dimensional, potentially non-smooth optimization problems and various cross-validation loops needed to optimize the parameters; moreover, permutation testing is necessary to assess the statistical significance of the results of such procedures in the absence of analytical tests. Multivariate statistical methods require thus many efforts to be tractable for this problem on both the algorithmic and implementation side, including the design of adapted dimension reduction schemes. Working in a distributed context is necessary to deal efficiently with the memory and computational loads.

Today, researchers have access to many computing capabilities to perform data-intensive analysis. The cloud is increasingly used to run such scientific applications, as it offers a reliable, flexible, and easy to use processing pool (Vaquero et al., 2008; Jackson et al., 2010; Hiden et al., 2012; Juve et al., 2012). The MapReduce paradigm (Chu et al., 2006; Dean and Ghemawat, 2008) is the natural candidate for these applications, as it can easily scale the computation by applying in parallel an operation on the input data (map) and then combine these partials results (reduce). However, some substantial challenges still have to be addressed to fully exploit the power of cloud infrastructures, such as data access, as it is currently achieved through high latency protocols, which are used to access the cloud storage services (e.g., Windows Azure Blob). To sustain geographically distributed computation, the storage system needs to manage concurrency, data placement and inter-site data transfers.

We propose an efficient framework that can manage inferences on neuroimaging-genetic studies with several phenotypes and permutations. It combines a MapReduce framework (TomusBLOB, Costan et al., 2013) with machine learning algorithms (Scikit-learn library) to deliver a scalable analysis tool. The key idea is to provide end-users the capability to easily describe the statistical inference that they want to perform and then to test the model on their own computers before running the very same code in the cloud at a larger scale. We illustrate the potential of our approach on real data with an experiment showing how the functional signal in subcortical brain regions of interest (ROIs) can be significantly predicted with genome-wide genotypes. In section 2, we introduce methodological prerequisites, then we describe our generic distributed machine learning approach for neuroimaging-genetic investigations and we present the cloud infrastructure. In section 3, we provide the description of the experiment and the results of the statistical analysis.

## 2. Materials and methods

### 2.1. Neuroimaging-genetic study

Neuroimaging-genetic studies test the effect of genetic variables on imaging target variables in presence of exogenous variables. The imaging target variables are activation images obtained through functional Magnetic Resonance Imaging (fMRI), that yield a standardized effect related to experimental stimulation at each brain location of a reference brain space. For a study involving *n* subjects, we generally consider the following model:

$$Y=X\beta_{1}+Z\beta_{2}+\epsilon ,$$

where ***Y*** is a *n* × *p* matrix representing the signal of *n* subjects described each by *p* descriptors (e.g., voxels or ROIs of an fMRI contrast image), ***X*** is the *n* × *q*_1_ set of *q*_1_ explanatory variables and ***Z*** the *n* × *q*_2_ set of *q*_2_ covariates that explain some portion of the signal but are not to be tested for an effect. ***β_1_*** and ***β_2_*** are the fixed coefficients of the model to be estimated, and ***ϵ*** is some Gaussian noise. ***X*** contains genetic measurements and variables in ***Z*** can be of any type (genetic, artificial, behavioral, experimental, …).

#### 2.1.1. The standard approach

It consists in fitting *p* Ordinary Least Square (OLS) regressions, one for each column of Y, as a target variable, and each time perform a statistical test (e.g., *F*-test) and interpret the results in term of significance (*p*-value). This approach suffers from some limitations. First, due to a low signal-to-noise ratio and a huge number of tests, this approach is not sensitive. Moreover, the statistical score only reflects the univariate correlation between a target and a set of *q*_1_ explanatory variables, it does not inform on their predictive power when considered jointly. Secondly, with neuroimaging data as a signal, we are not in a *case vs. control* study. It raises the question whether the variability in a population can be imputed to few rare genetic variants or if it is the addition of many small effects of common variants. Unfortunately, the model holds only if *n* » (*q*_1_ + *q*_2_), which is not the case with genome-wide genotypes.

#### 2.1.2. Heritability assessment

The goal of our analysis is to estimate the proportion of differences in a trait between individuals due to genetic variability. Heritability evaluation traditionally consists in studying and comparing homozygous and dizygous twins, but recently it has been shown that it can be estimated using genome-wide genotypes (Lee et al., 2011; Lippert et al., 2011; Yang et al., 2011b). For instance, common variants are responsible of a large portion of the heritability of human height (Yang et al., 2010) or schizophrenia (Lee et al., 2012). These results show that the variance explained by each chromosome is proportional to its length. As we consider fMRI measurements in an unsupervised setting (no disease), this suggests to use regression models that do not enforce sparsity. Like the standard approach, heritability has some limitations. In particular, the estimation of heritability requires large sample sizes to have an acceptable standard error (at least 4000 according to Lee et al., 2012). Secondly, the heritability is the ratio between the variance of the trait and the genetic variance in a population. Therefore, for a given individual, a trait with an heritability at 0.6 does not mean it can be predicted at 60% on average with the genotype. It means that a fraction of the phenotype variability is simply explained by the average genetic structure of the population of interest.

#### 2.1.3. High-dimensional statistics

The key point of our approach is to fit a model on training data (train set) and evaluate its goodness on unseen data (test set). To stabilize the impact of the sets for training and testing, a cross-validation loop is performed, yielding an average prediction score over the folds. This score yields a statistic value and a permutation test is performed to tabulate the distribution of this statistic under the null hypothesis and to estimate its significance (*p*-value). In practice, this corresponds to swapping the labels of the observations. As a prediction metric we generally choose the coefficient of determination (*R*^2^), which is the ratio between the variance of the prediction and the variance of the phenotypes in the test set. If we consider all the genotypes at the same time, this approach is clearly related to *heritability*, but focuses on the predictive power of the model and its significance. Through cross-validation, the estimation of the *CV*−*R*^2^ with an acceptable standard error does not require as large sample sizes as for the estimation of heritability (Yang et al., 2011a).

$$CV\text{-}R^{2}=1-mean_{(train,test)\in \text{split(n)}}\frac{\|Y^{test}-X^{test}\beta_{1}{}^{train}-Z^{test}\beta_{2}{}^{train}{\|}^{2}}{\|Y^{test}-Z^{test}\beta_{2}{}^{train}{\|}^{2}}$$

### 2.2. Generic procedure for distributed machine learning

If one just wants to compute the prediction score for few phenotypes, a multicore machine should be enough. But, if one is interested in the significance of this prediction score, one will probably need a computers farm (cloud, HPC cluster, etc.) Our approach consists in unifying the description and the computation for neuroimaging-genetic studies to scale from the desktop computer to the supercomputing facilities. The description of the statistical inference is provided by a descriptive configuration in human-readable and standard format: JSON (JavaScript Object Notation). This format requires no programming skills and is far easier to process as compared to the XML (eXtensible Markup Language) format. In a sense, our approach extends the Scikit-learn library (cf. next paragraph) for distributed computing, but focuses on a certain kind of inferences for neuroimaging-genetic studies. The next paragraphs describe the strategy, framework and implementation used to meet the heritability assessment objective.

#### 2.2.1. Scikit-learn

**Figure 1:**
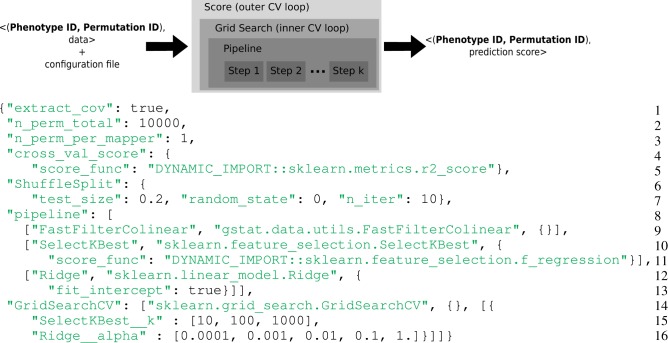
**Top**: Representation of the computational framework: given the data, a permutation and a phenotype index together with a configuration file, a set of computations are performed, that involve two layers of cross-validation for setting the hyper-parameters and evaluate the accuracy of the model. This yields a statistical score associated with the given phenotype and permutation. **Bottom**: Example of complex configuration file that describes this set of operations. *General parameters (Lines 1–3)*: The model contains covariates, the permutation test makes 10,000 iterations and only one permutation is performed in a task. *Prediction score (Lines 4–7)*: The metrics for the cross-validated prediction score is *R*^2^, the cross-validation loop makes 10 iterations, 20% of the data are left out for the test set and the seed of the random generator was set to 0. *Estimator pipeline (Lines 8–13)*: The first step consists in filtering collinear vectors, the second step selects the *K* best features and the final step is a ridge estimator. *Parameters selection (Lines 14–16)*: Two parameters of the estimator have to be set: the *K* for the *SelectKBest* and the *alpha* of the *Ridge* regression. A set of 3 × 5 parameters are evaluated.

Scikit-learn is a popular machine learning library in Python (Pedregosa et al., 2011) designed for a multicore station. In the Scikit-learn vocabulary, an estimator is an object that implements a fit and a predict method. For instance a Ridge object (lines 12–13 of Figure 1) is an estimator that computes the coefficients of the ridge regression model on the train set and uses these coefficients to predict data from the test set. If this object has a transform method, it is called a transformer. For instance a SelectKbest object (lines 10–11 of Figure 1) is a transformer that modifies the input data (the design matrix ***X***) by returning the *K* best explanatory variables w.r.t. a scoring function. Scikit-learn defines a Pipeline (lines 8–13 of Figure 1) as the combination of several transformers and an final estimator: It creates a combined estimator. Model selection procedures are provided to evaluate with a cross-validation the performance of an estimator (e.g., cross_val_score) or to select parameters on a grid (e.g., GridSearchCV).

#### 2.2.2. Permutations and covariates

Standard machine learning procedures have not been designed to deal with covariates (such as those assembled in the matrix ***Z***), which have to be considered carefully in a permutation test (Anderson and Robinson, 2001). For the original data, we fit an Ordinary Least Square (OLS) model between ***Y*** and ***Z***, then we consider the residuals of the regression (denoted *R_Y|Z_*) as the target for the machine learning estimator. For the permutation test, we permute *R_Y|Z_* (the permuted version is denoted R_Y|Z_^*^), then we fit an OLS model between R_Y|Z_^*^ and ***Z***, and we consider the residuals as the target for the estimator (Anderson and Robinson, 2001). The goal of the second OLS on the permuted residuals is to provide an optimal approximation (in terms of bias and computation) of the exact permutation tests while working on the reduced model.

#### 2.2.3. Generic problem

We identify a scheme common to the different kinds of inference that we would like to perform. For each target phenotype we want to compute a prediction score in the presence of covariates or not and to evaluate its significance with a permutation test. Scikit-learn algorithms are able to execute on multiple CPU cores, notably cross-validation loop, so a task will be executed on a multicore machine: cluster nodes or multicore virtual machine (VM). As the computational burden of different machine learning algorithms is highly variable, owing to the number of samples and the dimensionality of the data, we thus have to tune the number of tasks and their average computation time. An optimal way to tune the amount of work is to perform several permutations on the same data in a given task to avoid I/O bottlenecks. Finally, we put some constraints on the description of the machine learning estimator and the cross validation scheme:

- The prediction score is computed using the Scikit-learn cross_val_score function and the folds for this cross validation loop are generated with a ShuffleSplit object.
- An estimator is described with a Scikit-learn pipeline with one or more steps.
- Python can dynamically load modules such that a program can execute functions that are passed in a string or a configuration file. To notify that a string contains a Python module and an object or function to load, we introduce the prefix DYNAMIC_IMPORT::
- To select the best set of parameters for an estimator, model selection is performed using Scikit-learn GridSearchCV and a 5-folds inner cross-validation loop.

#### 2.2.4. Full example (cf. script in Figure 1)

- *General parameters (Lines 1–3)*: The model contains covariates, the permutation test makes 10,000 iterations and only one permutation is performed in a task. 10,000 tasks per brain target phenotypes will be generated.
- *Prediction score (Lines 4–7)*: The metrics for the cross-validated prediction score is *R*^2^, the cross-validation loop makes 10 iterations, 20% of the data are left out for the test set and the seed of the random generator was set to 0.
- *Estimator pipeline (Lines 8–13)*: The first step consist in filtering collinear vectors, the second step selects the *K* best features and the final step is a ridge estimator.
- *Parameters selection (Lines 14–16)*: Two parameters of the estimator have to be set: the *K* for the SelectKBest and the *alpha* of the Ridge regression. A set of 3 × 5 parameters are evaluated.

### 2.3. The cloud computing environment

Although researchers have relied mostly on their own clusters or grids, clouds are raising an increasing interest (Jackson et al., 2010; Simmhan et al., 2010; Ghoshal et al., 2011; Hiden et al., 2012; Juve et al., 2012). While shared clusters or grids often imply a quota-based usage of the resources, those from clouds are owned until they are explicitly released by the user. Clouds are easier to use since most of the details are hidden to the end user (e.g., network physical implementation). Depending on the characteristics of the targeted problem, this is not always an advantage (e.g., collective communications). Last but not least, clouds avoid owning expensive infrastructures—and associated high cost for buying and operating—that require technical expertise.

The cloud infrastructure is composed of multiple data centers, which integrate heterogeneous resources that are exploited seamlessly. For instance, the Windows Azure cloud has five sites in United States, two in Europe and three in Asia. As resources are granted *on-demand*, the cloud gives the illusion of infinite resources. Nevertheless, cloud data centers face the same load problems (e.g., workload balancing, resource idleness, etc.) as traditional grids or clusters.

In addition to the computation capacity, clouds often provide data-related services, like object storage for large datasets (e.g., S3 from Amazon or Windows Azure Blob) and queues for short message communication.

### 2.4. Neuroimaging-genetics computation in the cloud

In practice, the workload of the A-Brain application^1^ is more resource demanding than the typical cloud applications and could induce two undesirable situations: (1) other clients do not have enough resource to lease on-demand in a particular data center; (2) the computation creates performance degradations for other applications in the data center (e.g., by occupying the network bandwidth, or by creating high number of concurrent requests on the cloud storage service). Therefore, we divide the workload into smaller sub-problems and we select the different datacenters in collaboration with the cloud provider.

For balancing the load of the A-Brain application, the computation was distributed across four *deployments* in the two biggest Windows Azure datacenters. In the cloud context, a *deployment* denotes a set of leased resources, which are presented to the user as a set of uniform machines, called *compute nodes*. Each deployment is independent and isolated from the other deployments. When a compute node starts, the user application is automatically uploaded and executed. The compute nodes of a deployment belong to the same virtual private network and communicate with the outside world or other deployments either through *public endpoints* or using the cloud storage services (i.e., Windows Azure Blob or Queue).

**Figure 2:**
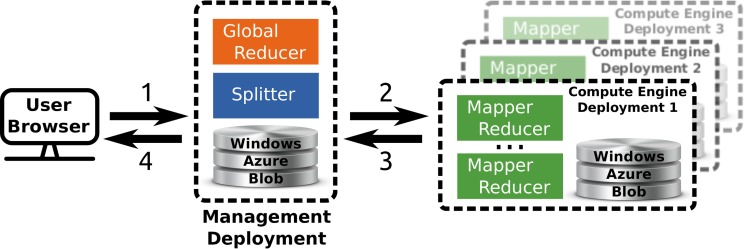
**Overview of the multi site deployment of a hierarchical Tomus-MapReduce compute engine**. **(1)** The end-user uploads the data and configures the statistical inference procedure on a webpage. **(2)** The *Splitter* partitions the data and manages the workload. The compute engines retrieves job information trough the Windows Azure Queues. **(3)** Compute engines perform the *map* and *reduce* jobs. The management deployment is informed of the progression via the Windows Azure Queues system and thus can manage the execution of the *global reducer*. **(4)** The user downloads the results of the computation on the webpage of the experiment.

TomusBlobs (Costan et al., 2013) is a data management system designed for concurrency-optimized PaaS-level (Platform as a Service) cloud data management. The system relies on the available local storage of the compute nodes in order to share input files and save output files. We built a processing framework (called TomusMapReduce) derived from MapReduce (Chu et al., 2006; Dean and Ghemawat, 2008) on top of TomusBlobs, such that it leverages its benefits by collocating data with computation. Additionally, the framework is restricted to *associative* and *commutative* reduction procedures (Map-IterativeReduce model Tudoran et al., 2012) in order to allow efficient out-of-order and parallel processing for the reduce phase. Although MapReduce is designed for single cluster processing, the latter constraint enables straightforward geographically distributed processing. The hierarchical MapReduce (which is described in Costan et al., 2013) aggregates several deployments with *MapReduce engines* and a last deployment that contains a *MetaReducer*, that computes the final result, and a *Splitter*, that partitions the data and manages the overall workload in order to leverage data locality. Job descriptions are sent to the MapReduce engines via Windows Azure Queue and the MetaReducer collects intermediate results via Windows Azure Blob. For our application, we use the Windows Azure Blob storage service instead of TomusBlobs for several reasons: (1) concurrency-optimized capabilities are not relevant here; (2) for a very long run, it is better to rely on a proven storage; (3) TomusBlob storage does not support yet multi-deployments setting. An overview of the framework is shown in Figure 2.

For our application, the *Map* step yields a prediction score for an image phenotype and a permutation, while the *reduce* step consists in collecting all results to compute statistic distribution and corrected *p*-values. The reduce operation is trivially commutative and associative as it consists in searching the maximum of the statistic for each permutation (Westfall and Young, 1993). The upper part of Figure 1 gives an overview of the generic mapper.

### 2.5. Imagen: a neuroimaging-genetic dataset

IMAGEN is a European multi-centric study involving adolescents (Schumann et al., 2010). It contains a large functional neuroimaging database with fMRI associated with 99 different contrast images for 4 protocols in more than 2000 subjects, who gave informed signed consent. Regarding the functional neuroimaging data, we use the Stop Signal Task protocol (Logan, 1994) (SST), with the activation during a *[go wrong]* event, i.e., when the subject pushes the wrong button. Such an experimental contrast is likely to show complex mental processes (inhibition failure, *post-hoc* emotional reaction of the subject), that may be hard to disentangle. Our expectation is that the amount of Blood Oxygen-Level Dependent (BOLD) response associated with such events provides a set of global markers that may reveal some heritable psychological traits of the participants. Eight different 3T scanners from multiple manufacturers (GE, Siemens, Philips) were used to acquire the data. Standard preprocessing, including slice timing correction, spike and motion correction, temporal detrending (functional data) and spatial normalization (anatomical and functional data), were performed using the SPM8 software and its default parameters; functional images were resampled at 3mm resolution. All images were warped in the MNI152 coordinate space. Obvious outliers detected using simple rules such as large registration or segmentation errors or very large motion parameters were removed after this step. BOLD time series was recorded using Echo-Planar Imaging, with *TR* = 2200ms, *TE* = 30ms, flip angle = 75° and spatial resolution 3 × 3 × 3mm. Gaussian smoothing at 5mm-FWHM was finally added. Contrasts were obtained using a standard linear model, based on the convolution of the time course of the experimental conditions with the canonical hemodynamic response function, together with standard high-pass filtering procedure and temporally auto-regressive noise model. The estimation of the first-level was carried out using the SPM8 software. T1-weighted MPRAGE anatomical images were acquired with spatial resolution 1 × 1 × 1mm, and gray matter probability maps were available for 1986 subjects as outputs of the SPM8 *New Segmentation* algorithm applied to the anatomical images. A mask of the gray matter was built by averaging and thresholding the individual gray matter probability maps. More details about data preprocessing can be found in Thyreau et al. (2012).

DNA was extracted from blood samples using semi-automated process. Genotyping was performed genome-wide using Illumina Quad 610 and 660 chips, yielding approximately 600,000 autosomic SNPs. 477,215 SNPs are common to the two chips and pass *plink* standard parameters (Minor Allele Frequency >0.05, Hardy-Weinberg Equilibrium *P*<0.001, missing rate per SNP <0.05).

## 3. An application and results

### 3.1. The experiment

The aim of this experiment is to show that our framework has the potential to explore links between neuroimaging and genetics. We consider an fMRI contrast corresponding to events where subjects make motor response errors (*[go wrong]* fMRI contrast from a Stop Task Signal protocol). Subjects with too many missing voxels or with bad task performance were discarded. Regarding genetic variants, 477,215 SNPs were available. Age, sex, handedness and acquisition center were included in the model as confounding variables. Remaining missing data were replaced by the median over the subjects for the corresponding variables. After applying all exclusion criteria 1459 subjects remained for analysis. Analyzing the whole brain with all the genetic variants remains intractable due to the time and memory requirements and dimension reduction techniques have to be employed.

#### 3.1.1. Prior neuroimaging dimension reduction

In functional neuroimaging, brain atlases are mainly used to provide a low-dimensional representation of the data by considering signal averages within groups of neighboring voxels. In this experiment we focus on the subcortical nuclei using the Harvard–Oxford subcortical atlas. We extract the functional signal of 14 regions of interest, 7 in each hemisphere: thalamus, caudate, putamen, pallidum, hippocampus, amygdala and accumbens (see Figure 4). White matter, brain stem and ventricles are of no interest for functional activation signal and were discarded. This prior dimension reduction decreases the number of phenotypes from more than 50,000 voxels to 14 ROIs.

#### 3.1.2. Configuration used (cf. script in Figure 3)

- *(Lines 1–3)*: covariates, 10,000 permutations and 5 permutations per computation unit (mapper).
- *(Lines 4–7)*: 10-folds cross-validated *R*^2^.
- *(Lines 9–11)*: The first step of the pipeline is an univariate features selection (*K* = 50,000). This step is used as a dimension reduction so that the next step fits in memory.
- *(Lines 12–13)*: The second and last step is the ridge estimator with a low penalty (*alpha* = 0.0001).

**Figure 3:**
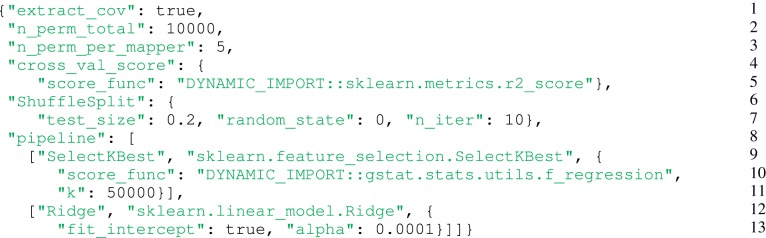
**Configuration used for the experiment**. *(Lines 1–3)*: Covariates, 10,000 permutations and five permutations per computation unit (mapper). *(Lines 4–7)*: 10-folds cross-validated *R*^2^. *(Lines 9–11)*: The first step of the pipeline is an univariate features selection (*K* = 50,000). This step is used as a dimension reduction so that the next step fits in memory. *(Lines 12–13)*: The second and last step is the ridge estimator with a low penalty (*alpha* = 0.0001).

The goal of the experiment described by this configuration file is to evaluate how the 50,000 mostly correlated genetic variants, once taken together, are predictive of each ROI and to associate a *p*-value with these prediction scores. Note that more than 50,000 covariates would not fit into memory. This configuration generates 28,000 map tasks (14 × 10,000/5), but we can set to 1 the number of permutations per task, which means that the computation can use up to 140,000 multicore computers in parallel, and thus millions of CPU cores.

#### 3.1.3. The cloud experimental setup

The experiment was performed using the Microsoft Windows Azure PaaS cloud in the North and West US datacenters, that were recommended by the Microsoft team for their capacity. We use the Windows Azure storage services (Blob and Queue) in both datacenters in order to take advantage of the data locality. Due to our memory requirements, the *Large VM* type (4 CPU cores, 7 GB of memory and 1000 GB of disk) is the best fit regarding the Azure VMs offer^2^.

#### 3.1.4. TomusBlobs

We set up two deployments in each of the two recommended sites for a total of four deployments. It used 250 large VM nodes, totalizing 1000 CPUs: each of the 3 MapReduce engines deployments had 82 nodes and the last deployment used 4 nodes. The reduction process was distributed in approximately 600 reduce jobs.

### 3.2. Results

#### 3.2.1. Cloud aspects

The experiment timespan was 14 days. The processing time for a single map job is approximately 2h. There are no noticeable time differences between the execution times of the map jobs with respect to the geographical location. In large infrastructures like the clouds, failures are possible and applications need to cope with this. In fact, during the experiment the Azure services became temporary inaccessible^3^, due to a failure of a secured certificate. Despite this problem, the framework was able to handle the failure with a fault tolerance mechanism which suspended the computation until all Azure services became available again. The monitoring mechanism of the *Splitter*, that supervises the computation progress, was able to restore aborted jobs. The IterativeReduce approach eliminates the implicit barrier between mappers and reducers, but yields negligible gains due to the huge workload of the mappers. The effective cost of the experiment was approximately equal to 210,000h of sequential computation, which corresponds to almost $20,000 (VM pricing, storage and outbound traffic).

#### 3.2.2. Application side

**Figure 4:**
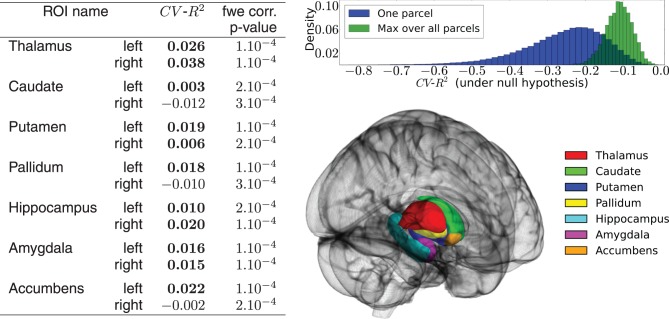
**Results of the real data analysis procedure**. (**Left**) predictive accuracy of the model measured by cross-validation, in the 14 regions of interest, and associated statistical significance obtained in the permutation test. (**Up right**) distribution of the *CV*−*R*^2^ at chance level, obtained through a permutation procedure. The distribution of the max over all ROIs is used to obtain the family-wise error corrected significance of the test. (**Bottom right**) outline of the chosen ROIs.

Figure 4 shows a summary of the results. Despite the fact that some prediction scores are negative, the activation signal in each ROI is fit significantly better than chance using the 50,000 best genetic variants over the 477,215. The mean BOLD signal is better predicted in the left and right thalamus. The distribution of the *CV*−*R*^2^ is also very informative, showing that by chance the mean prediction score is negative (familywise-error corrected or not). While this phenomenon is somewhat counter-intuitive within the framework of classical statistics, it should be pointed out that the cross-validation procedure used here opens the possibility of negative *R*^2^: this quantity is by definition a model comparison statistic that takes the difference between a regression model with a non-informative model; in high-dimensional settings, a poorly fitting linear model performs (much) worse than a non-informative model. Hence a model performing at chance gets a negative score: This is actually what happens systematically when the association between *y* and *X* is broken by the permutation procedure, even if we consider the supremum over many statistical tests (Westfall and Young, 1993). A slightly negative value can thus be the marker of a significant association between the variables of interest. Twin and SNP-based studies suggest high heritability of structural brain measures, such as total amount of gray and white matter, overall brain volume and addiction-relevant subcortical regions. Heritability estimates for brain measures are as high as 0.89 (Kremen et al., 2010) or even up to 0.96 (van Soelen et al., 2012) and subcortical regions appear to be moderately to highly heritable. One recent study on subcortical volumes (den Braber et al., 2013) reports highest heritability estimates for the thalamus (0.80) and caudate nucleus (0.88) and lowest for the left nucleus accumbens (0.44). Despite the fact that the *CV*−*R*^2^ metric is not exactly an heritability measurement, our metric evaluates the predictability of the fitted model (i.e., how well it predicts the activation signal of a brain region with genetic measurements on unseen data) which is a good proxy for heritability. Thus, our results confirm that brain activation signals are an heritable feature in subcortical regions. These experiments can be used as a basis to further localize the genetic regions (pathways or genes) that are actually predictive of the functional activation. An important extension of the present work is clearly to extend this analysis to the cortical regions.

## 4. Conclusion

The quantitative evaluation of statistical models with machine learning techniques represents an important step in the comprehension of the associations between brain image phenotypes and genetic data. Such approaches require cross validation loops to set the hyper-parameters and to evaluate performances. Permutations have to be used to assess the statistical significance of the results, thus yielding prohibitively expensive analyses. In this paper, we present a framework that can deal with such a computational burden. It relies on two key points: (1) it wraps the Scikit-learn library to enable coarse grain distributed computation. Yet it enforces some restrictions, i.e., it solves only a given class of problems (pipeline structure, cross-validation procedure and permutation test). The result is a simple generic code (few lines) that provides the user a quick way to conduct early, small-scale investigations on its own computer or at a larger scale on a high-performance computing cluster. With JSON we provide a standard format for the description of statistical inference so that no programming skills are required and so that it can be easily generated from a webpage form. (2) TomusBLOB permits to execute seamlessly the very same code on the Windows Azure cloud. We could also disable some parts of TomusBLOB to achieve a good compromise between the capabilities and the robustness. We demonstrate the scalability and the efficiency of our framework with a 2 weeks geographically distributed execution on hundreds of virtual machines. The results confirm that brain activation signals are an heritable feature.

### Conflict of interest statement

The authors declare that the research was conducted in the absence of any commercial or financial relationships that could be construed as a potential conflict of interest.

## Footnotes

^1^http://www.irisa.fr/kerdata/abrain/

^2^http://msdn.microsoft.com/fr-fr/library/windowsazure/dn197896.aspx

^3^Azure Failure Incident: http://readwr.it/tAq
